# Supplementary material for: Resistance of Modern Russian Winter Wheat Cultivars to Yellow Rust
Source: Plants (Basel). 2023 Oct 3;12(19):3471. doi: 10.3390/plants12193471 (PMC10574662; doi:10.3390/plants12193471)
Supplement: Supplementary file 1 [file plants-12-03471-s001.zip › plants-2609884-supplementary.pdf]

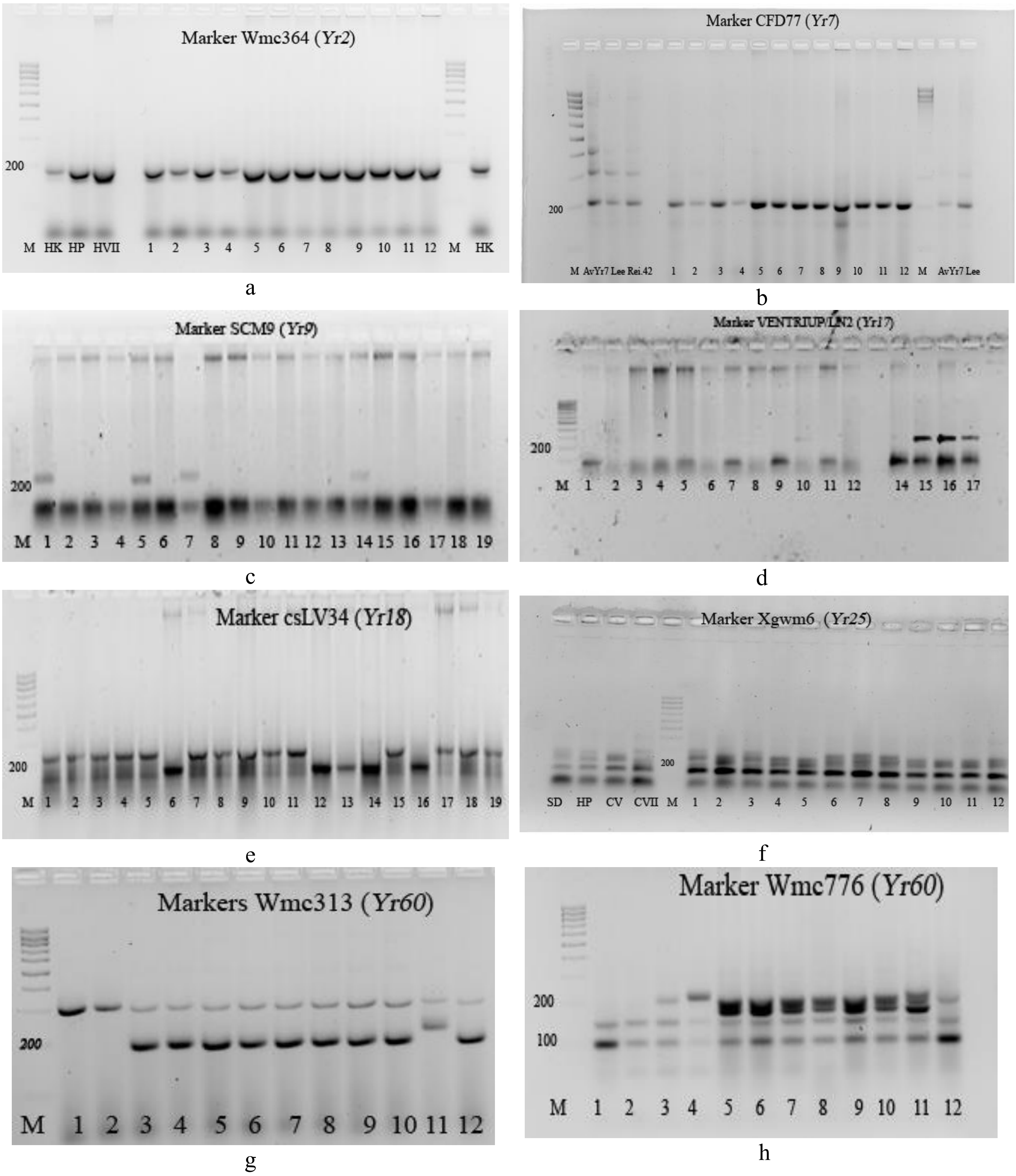

Fig. 1. Electrophoretogram for microsatellite markers of *Yr2, Yr7, Yr9, Yr17, Yr18, Yr25* and *Yr60* resistance genes.

M - DNA Ladder 100 bp (Dialat), HK - Heines Kolben, HP - Heines Peko, HVII - Heines VII, Rei.42 - Reichersberg 42, SD - Strubes Dickkopf,

a, b, f, g, h: 1 – Arsenal, 2 - Bazal't 2, 3 – Bodryy, 4 – Videya, 5 – Gerda, 6 – Donmira, 7 – Iridas, 8 – Kavalerka, 9 – Korona, 10 – Markiz, 11 - Stat', 12 - STRG 8060 15

c: 1) Ambar, , 2) Agrofak 100, 3) Batya, 4) Vladi, 5) Leo, 6) Volodya, 7) Zarechnaya, 8) Izaura, 9) Podruga, 10) En Mars, 11) Mig, 12) Morets, 13) Pal'mira 18, 14) Fyodor, 15) Sirena, 16) Studencheskaya niva, 17) Taya, 18) Timiryazevskaya yubileynaya, 19) En Foton.

d: 1) Arsenal, 2) Bazal't 2, 3) Bodryy, 4) Videya, 5) Gerda, 6) Donmira, 7) Iridas, 8) Kavalerka, 9) Korona, 10) Markiz, 11) Stat', 12) STRG 8060 15, 13) Timiryazevka 150, 14) Nemchinovskaya 85, 15) Gomer, 16) Thatcher Lr37

e: 1) Agrofak 100, 2) Ambar, 3) Batya, 4) Vladi, 5) Volodya, 6) En Foton, 7) Zarechnaya, 8) Izaura, 9) Leo, 10) En Mars, 11) Mig, 12) Morets, 13) Pal'mira 18, 14) Podruga, 15) Sirena, 16) Studencheskaya niva, 17) Taya, 18) Timiryazevskaya yubileynaya, 19) Fyodor.
